# Punch-excised explants of bovine mammary gland to model early immune response to infection

**DOI:** 10.1186/s40104-023-00899-0

**Published:** 2023-07-07

**Authors:** Pablo Gomes Noleto, Florence B. Gilbert, Christelle Rossignol, Patricia Cunha, Pierre Germon, Pascal Rainard, Rodrigo Prado Martins

**Affiliations:** ISP, INRAE, Université de Tours, UMR1282, Nouzilly, France

**Keywords:** Bovine teat, *Escherichia coli*, Explant, Mastitis, *Staphylococcus aureus*, 3Rs

## Abstract

**Background:**

Mammary gland (MG) infections (mastitis) are frequent diseases of dairy cows that affect milk quality, animal welfare and farming profitability. These infections are commonly associated with the bacteria *Escherichia coli* and *Staphylococcus aureus*. Different in vitro models have been used to investigate the early response of the MG to bacteria, but the role of the teat in mastitis pathogenesis has received less attention. In this study, we used punch-excised teat tissue as an ex vivo model to study the immune mechanisms that arise early during infection when bacteria have entered the MG.

**Results:**

Cytotoxicity and microscopic analyses showed that bovine teat sinus explants have their morphology and viability preserved after 24 h of culture and respond to ex vivo stimulation with TLR-agonists and bacteria. LPS and *E. coli* trigger stronger inflammatory response in teat when compared to LTA and *S. aureus*, leading to a higher production of IL-6 and IL-8, as well as to an up-regulation of proinflammatory genes. We also demonstrated that our ex vivo model can be applied to frozen-stored explants.

**Conclusions:**

In compliance with the 3Rs principle (replacement, reduction and refinement) in animal experimentation, ex vivo explant analyses proved to be a simple and affordable approach to study MG immune response to infection. This model, which better reproduces organ complexity than epithelial cell cultures or tissue slices, lends itself particularly well to studying the early phases of the MG immune response to infection.

**Supplementary Information:**

The online version contains supplementary material available at 10.1186/s40104-023-00899-0.

## Background

Mastitis is an inflammation of udder tissue mainly caused by bacteria, which is considered to be one of the most frequent and expensive diseases in the dairy industry. It has been estimated that mastitis costs up to $2 billion per year for the US dairy industry alone, and up to €198 per cow at the farm level in Europe [1–3]. Besides affecting milk production and quality, these infections also represent a concern for human health as potential sources of foodborne pathogens and antibiotic-resistant bacteria spread [4, 5].

Infections by *Escherichia coli* and *Staphylococcus aureus,* two of the major pathogens involved in bovine mastitis, differ in their pathophysiology and clinical manifestation: *E. coli* provokes clinical mastitis and elicits acute inflammation which may result in death or extensive damage to mammary tissues, whereas *S. aureus* infections often start with an acute phase that leads to chronic subclinical inflammation [6–9]. These infections also differ from an epidemiological point of view: *S. aureus* causes contagious mastitis, the infected mammary quarters being the major reservoir of bacteria, whereas *E. coli* leads to environmental mastitis associated with bacterial strains whose primary source is the environment of affected cows [10, 11].

To date, most of knowledge on immune response to mastitis is based on observations from in vivo experimental and field infections [12–14]. Nevertheless, the ethical impact of animal experimentation has gained importance worldwide and more efforts need to be undertaken for an effective implementation of the 3Rs (reduction, refinement and replacement) principle in the use of animals for research purposes [3, 15].

Various alternatives to animal use in science have been suggested [16], but their implementation in an effective manner is still necessary, especially for studies focused on livestock species. Among them, ex vivo tissue analysis represents an attractive option, as this approach has as advantage enabling the in situ study of mechanisms involved in cell–cell interactions, cell signalling, immune response and tissue physiology [17, 18]. Additionally, ex vivo models can be successfully based on slaughter industry waste, characterizing a low cost and accessible method.

Previous studies addressing the immune response of bovine mammary gland (MG) to infections have mostly targeted lobule-alveolar tissue and epithelial cells [8, 9, 17–19]. Mastitis-causing pathogens invade the MG via the teat canal, then spread through the teat sinus, the gland cistern and the large ducts to disseminate in the MG. The earliest signs of inflammation are detectable in the teat sinus tissue [20, 21], which is armed of anatomical and physiological features considered as the first protecting barrier to infections [22, 23]. Thus, the immune response mechanisms that take place in this part of the udder deserves a particular attention.

In this study, we set up an ex vivo model to study the innate immune response of bovine teats to two major mastitis-causing pathogens. Our results indicate that teat tissue explants are particularly well suited to study the early phase of the inflammatory immune response to mammary infections and represent a helpful alternative to in vivo approaches for the study of host response to mastitis.

## Material and methods

### Preparation of explants

Teats were collected from MGs with no signs of disease from 28 slaughtered dairy cows in a French commercial abattoir. Before sampling, udder skin was cleaned with alcohol-soaked paper towels and teats were kept in a Hank’s balanced salt solution (HBSS, H6648, Sigma-Aldrich, Irvine, United Kingdom) with antibiotics (100 IU/mL penicillin combined with 100 µg/mL streptomycin and 0.25 μg/mL amphotericin B (A5955, Sigma-Aldrich, Saint Louis, USA)) until further processing (< 2 h).

**Fig. 1:**
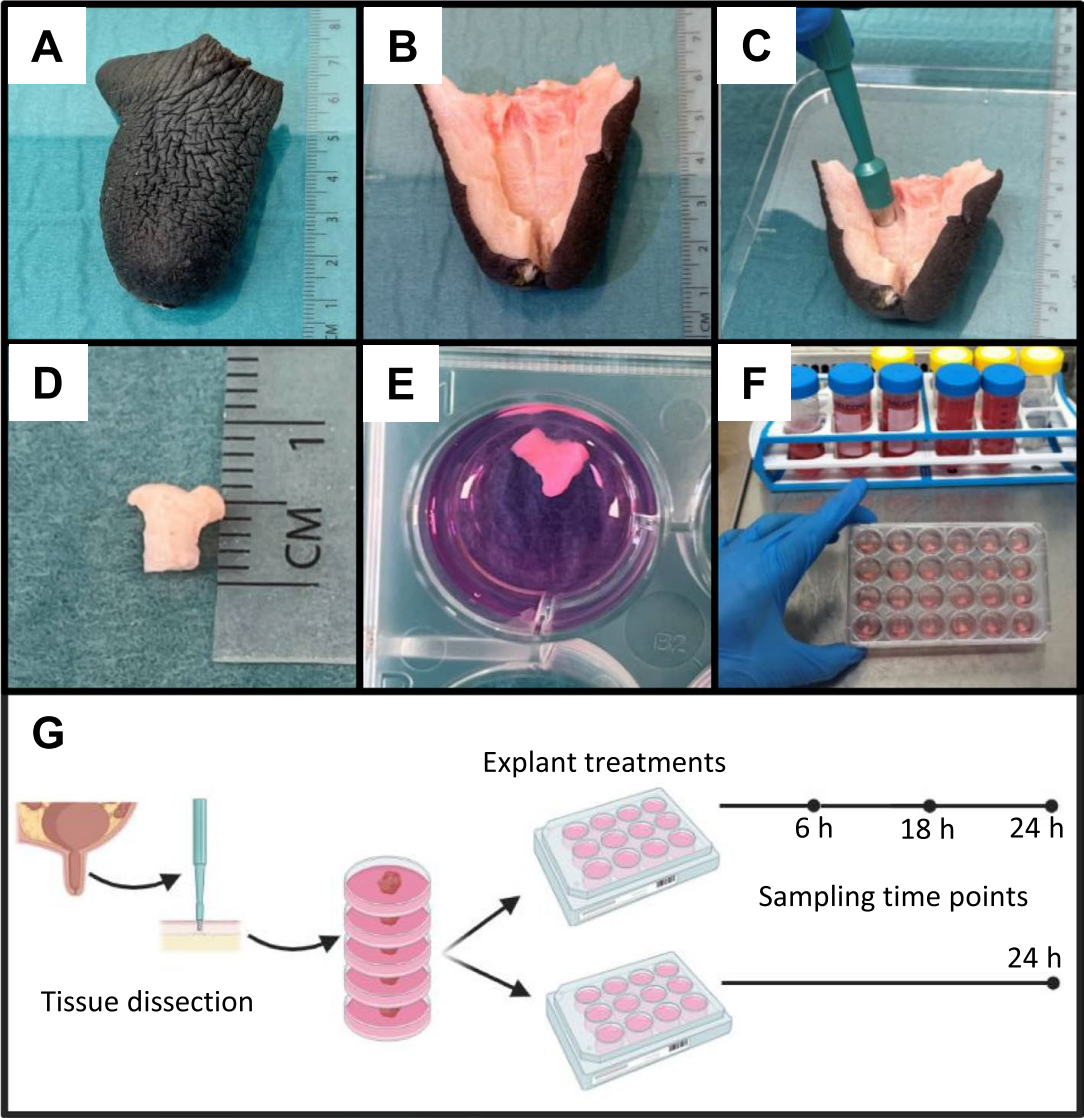
Collection of bovine teat explants for ex vivo cultures. **A** Intact teat. **B** Dissected teat. **C** Collection of explants using a 6-mm diameter sterile biopsy punch. **D** Teat explant. **E**–**F** Ex vivo stimulation of explants. **G** Cartoon depicting tissue and supernatant sampling strategies

Teats were subsequently dissected under sterile conditions in a class II biological safety cabinet (Fig. 1A–B) and washed with Dulbecco’s phosphate-buffered saline solution (D-PBS, D8537, Sigma-Aldrich, Irvine, United Kingdom) supplemented with 100 IU/mL penicillin, 100 µg/mL streptomycin and 0.25 μg/mL amphotericin B. Explants (about 50 mg) were then collected from the mucosal surface of teat sinus using sterile 6-mm-diameter biopsy punches (LCH-PUK-60, Kai Medical, Solingen, Germany) (Fig. 1C–D) and incubated in HBSS supplemented with 100 IU/mL penicillin, 100 µg/mL streptomycin and 0.25 µg/mL amphotericin B for 5 min. Afterwards, explants were individually transferred to 24-well tissue culture plates (353047, Falcon – Corning, Durham, USA) containing 1 mL of complete medium (Advanced DMEM F12 (12634010, Gibco, Carlsbad, USA)) supplemented with 10% heat-inactivated endotoxin-free foetal bovine serum (F7524, Sigma-Aldrich, Brazil Origin, Saint Louis, USA), 2 mmol/L glutamine (25030, Gibco, Paisley, United Kingdom), 100 IU/mL penicillin combined with 100 µg/mL streptomycin, and 0.25 µg/mL amphotericin B (Fig. 1E–F), and cultured in a humidified atmosphere with 5% CO_2_ in air at 37 °C until treatments (< 1 h).

### Explant treatments

Toll-like receptor (TLR) agonists: explants were exposed to 10, 1 and 0.1 µg/mL of *E. coli* 0111 ultra-pure lipopolysaccharide (LPS, tlrl-3pelps, InvivoGen, San Diego, USA) or 10 and 1 µg/mL of *S. aureus* lipoteichoic acid (LTA, tlrl-pslta, InvivoGen, San Diego, USA). Before treatments, medium was removed and replaced with 1 mL of complete medium alone (control) or containing the tested molecules. Tissue response to treatment was firstly assessed in a kinetics analysis as follows: culture medium was harvested at 6, 18 and 24 h and replaced with fresh complete medium containing the same TLR agonist concentration in order to estimate the cytokine release at 0–6, 6–18 and 18–24 h intervals. In a second analysis, explants were treated in 2 mL of complete medium and supernatants were harvested at 24 h after treatments (Fig. 1G).

Bacteria: *E. coli* (strain P4) [24] and *S. aureus* (strain D4 169–32) [25] isolates were cultured overnight at 37 °C in 10 mL brain heart infusion (CM1136, Oxoid, Hampshire, United Kingdom) medium. Then, 200 µL of culture were re-inoculated in BHI and cultured for 3.5 h. Cultures were centrifuged (3,500 × *g* for 15 min), bacterial pellets were resuspended with 1 mL DPBS and heat-inactivated at 60 °C for 35 min in a water bath. Inactivated bacterial suspension was subsequently centrifuged (10,000 × *g* for 5 min), washed two times with DPBS and resuspended in complete medium at a dilution of 1 × 10^7^ CFU/mL, according to the OD at 600 nm. For explants stimulation, medium was removed and replaced with 1 mL of bacterial suspension. Supernatants were collected as described for treatments with TLR-agonists.

In all treatments, explants obtained from the same teat were used in all tested conditions and at least one teat from four different animals was used.

### Histological examination

After treatments, explants were washed twice with DPBS and fixed with 4% paraformaldehyde (158127, Sigma-Aldrich, Saint Louis, USA) for 48 h at room temperature. Fixed samples were kept in DPBS at 4 °C (< 1 week) and embedded in paraffin under standard conditions. Tissue sections of 5 μm thick were cut, collected onto treated glass slides (SuperFrost Plus, 6319483, Thermo Scientific, Waltham, USA), dried for 2 d at 37 °C and then overnight at 56 °C. Paraffin-embedded samples were then stained with haematoxylin and eosin (H&E). Representative photomicrographs were collected using a fluorescence microscope (Eclipse 80i Nikon, Tokyo, Japan) and images were processed using Image J [26].

### Lactate dehydrogenase (LDH) assay

In order to evaluate integrity of explants under the tested culture conditions, lactate dehydrogenase (LDH) release was measured as described elsewhere [27]. Briefly, supernatants were harvested after treatments and explants were mixed with lysis buffer [5 mmol/L EDTA, 150 mmol/L NaCl, 50 mmol/L Tris–HCl (pH 7.4), 1% Triton, anti-proteases (1 tablet/50 mL buffer, Protease inhibitor cocktail tablets, O4693132001, Roche, Mannhein, Germany)] in a lysing matrix D tube (6913500, MP Biomedicals, Thuringer, Germany). Samples were then homogenized in a Fast-Prep device (6004500, MP Biomedicals, Solon, USA) as follows: 2 cycles of 6 m/s for 35 s with a 5 min break in between. The concentration of LDH in tissue and supernatant was assessed using the non-radioactive Cytotoxicity Assay kit (G1780, Promega, Madison, USA) according to the manufacturer’s instructions. The cytotoxicity percentage was calculated according to the following formula: cytotoxicity (%) = [(OD_490_ of LDH in the supernatant)/(OD_490_ of LDH in the supernatant + OD_490_ of LDH in the explant homogenate)] × 100. Explants cultured under starvation-conditions (DPBS only) were used as positive control.

### Enzyme-linked immunosorbent assay (ELISA)

The release of IL-6 and IL-8 in culture supernatants was measured by ELISA using commercially available kits (Bovine IL-8 ELISA Flex 3114-1H-20 Mabtech, Nacka Strand, Sweden; Bovine IL-6 Do-It-Yourself ELISA DIY0670B-003 Kingfisher Biotech, Saint Paul, USA) according to the manufacturer’s instructions. In order to consider differences in explants weight, cytokines concentrations are reported in picogram per milligram of tissue.

### Gene expression analysis

Explants were placed in 1.5-mL tubes containing 500 µL of RNA Later (AM7024, ThermoFisher, Vilnius, Lithuania), incubated overnight at 4 °C and stored at −80 °C until use. Total RNA was extracted by homogenising tissue in 2-mL tubes containing 0.8 mL of TRI Reagent (AM9378, ThermoFisher, Vilnius, Lithuania) and lysing matrix D at a rate of 6 m/s for 1 min, 5 min break and 6 m/s for 30 s using a FastPrep 24. After homogenisation, tubes were centrifuged at 12,000 × *g* for 10 min and the supernatants were processed using Nucleospin RNA isolation kit (740955, Macherey–Nagel, Duren, Germany) according to the manufacturer’s instructions. RNA was then reverse-transcribed with iScriptTM Reverse Transcriptase mix (1708841, Biorad, Hercules, USA) according to the manufacturer’s conditions and reverse transcription-quantitative PCR assays were performed in a LightCycler 480 instrument (Roche). Four microliter of 10-fold diluted cDNA were added to a mixture of (2×) iTaq Universal SYBR Green Supermix (1708887, Biorad, Hercules, USA) and 0.25 μmol/L of each primer in a total volume of 10 μL. Thermal protocol was 95°C for 5 min followed by 40 cycles of 95 °C for 10 s, 60 °C for 30 s and acquisition of a melting curve at the end of the run. The specificity of primer pairs was checked via melting curve analysis. Primers used in this study (Additional file 3: Table S1) were designed and tested as previously described [28]. Fold changes were calculated by the ΔΔCt method using ACTB, PPIA, GAPDH as reference genes [29].

### Flow cytometry

For the production of single cell suspensions, explants were manually minced with a scalpel blade, mixed with digestion buffer [25 mg/mL collagenase IV (LS004188, Worthington, Lakewood, USA), 50 mg/mL BSA (P061391500, Pan Biotech, USA), 10 mg DNAse I (11284932001, Roche, Mannheim, Germany) and 5 mL of Tryple (12604–013, Gibco, Grand Island, USA) in 50 mL of HBSS] and incubated for 1 h in an orbital shaker (100 r/min) at 37 °C. Alternative methods for tissue digestion were tested and are described in the Supplementary Fig. S1 and S2.

Tissue digestions were filtered through a 100-μm cell strainer (352360, Falcon, Durham, USA), centrifuged at 400 × *g* for 10 min and cell pellets were washed twice with FACS buffer (DPBS without Ca^2^^+^ and Mg^2^^+^ supplemented with 2% (v/v) normal goat serum (16210–072, Gibco, Auckland, New Zealand) and 2 mmol/L EDTA. For surface markers staining, cells were processed as described by Cunha et al. [30] using the following antibodies: Anti-bovine G1 (WSC0608B, clone CH138A, KingFisher, Saint Paul, USA), anti-sheep CD45 conjugated to FiTC (MCA832F, clone CC1, BioRad, USA) and anti-human CD14 conjugated to Alexa 647 (MCA1568A647, clone TUK4, BioRad, USA). Anti-mouse IgM conjugated to PE-Cy7 (406513, clone RMM-1, Biolegend, San Diego, USA) was used as secondary antibody. Incubations with conjugated primary antibodies were carried out after primary and secondary antibodies. Dead cells were labelled using the 450 Fixable Viability (65086314, eFluor, eBioscience, Carlsband, USA) after antibody incubations. Cells were examined using a BD LSR Fortessa™ X-20 cytometer (BD Biosciences, New Jersey, USA) and Kaluza software (Beckman Coulter, Brea, USA) was used for data analysis.

### Tissue freezing protocol

Explants were placed into 1.8-mL cryotubes containing 1 mL of freezing medium [10% DMSO (D2438, Sigma-Aldrich, Irvine, United Kingdom)] in foetal bovine serum, frozen at −80 °C in a CoolCell alcohol-free freezer container (Thermo Scientific, Waltham, USA) for 48 h and then kept at −80 °C in conventional storage boxes until processing (< 1 month). For thawing, cryotubes were individually incubated at 37 °C in a water bath and explants were gently washed with 1 mL of pre-warmed complete medium before been completely defrosted. Samples were twice washed with medium for DMSO removal and processed as described for fresh tissue.

### Data analysis

Data were analysed and plotted using GraphPad Prism, version 6.0 (GraphPad Software Incorporation, Boston, USA). Kruskal–Wallis test was used to compare independent groups and pairwise comparisons were carried out using the Dunn’s test. Data shown represent the minimum and maximum values, as well as the sample median, the first and third quartiles from at least four individual replicates (four teats from four different cows).

## Results

### Teat explants are viable, show normal tissue architecture and respond to ex vivo stimulation with TLR-agonists

**Fig. 2:**
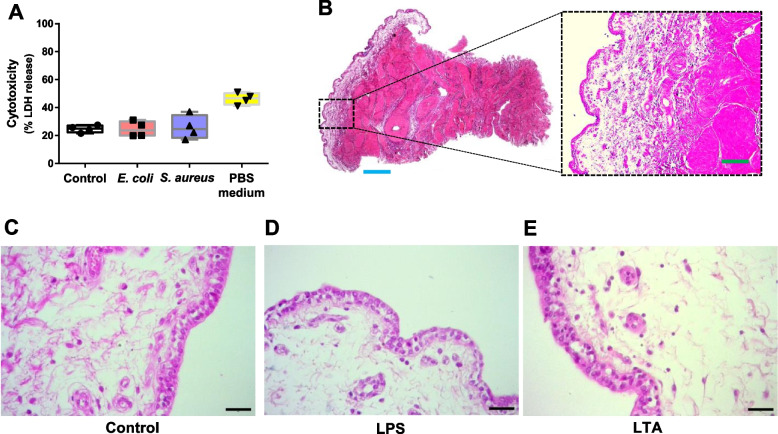
Tissue integrity upon stimulation with TLR-agonists. **A** Evaluation of cytotoxicity by lactate dehydrogenase (LDH) release assay. Explants were treated with LPS (1 µg/mL) or LTA (10 µg/mL) for 24 h. Complete medium alone and DPBS (nutrient starvation) were used as negative and positive controls, respectively. Box-plot shows data from 4 independent replicates (4 animals, 1 teat/animal). **B** Representative photomicrograph of an explant stained with haematoxylin and eosin. Dashed rectangle highlights typical teat microscopic architecture. **C–E** Histological analysis of explants stimulated as described in **A**. Blue bar = 500 µm. Green bar = 50 µm. Black bars = 20 µm

**Fig. 3:**
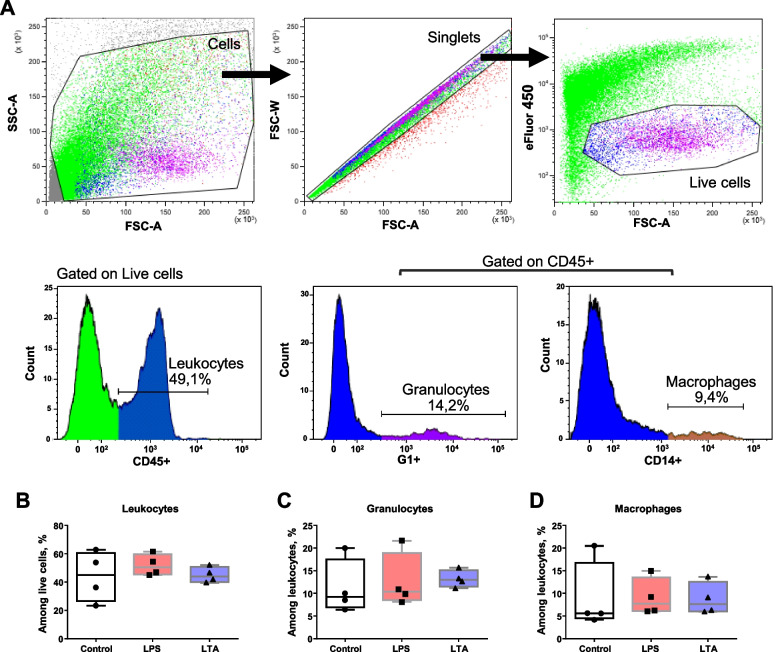
Analysis of cell populations in teat explants after treatment with TLR-agonists. **A** Gating strategy set up to analyse leukocyte populations by flow cytometry. **B–D** Explants were treated with LPS (1 µg/mL) or LTA (10 µg/mL) for 24 h. Complete medium alone was used as control. Percentages of leukocytes (**B**), granulocytes (**C**) and macrophages (**D**). Box-plots show data from 4 independent replicates (4 animals, 1 teat/animal)

Initially, we took advantage of the LDH release assay to verify that teat explants kept in culture were viable and could be used for further analysis. As shown in Fig. 2A, no differences in cytotoxicity levels were observed among control and treated explants. In all tested conditions, the median percentage of cytotoxicity remained below 25% and was significantly inferior to the levels observed for nutrient-starved tissue (PBS only). In addition, histological analyses demonstrated that explants show a normal tissue architecture upon 24 h culture, which is characterized by the presence of a double layered epithelium sustained by stromal tissue containing blood vessels and surrounded by a muscular tissue layer (Fig. 2B). A higher magnification view of tissue showed the absence of remarkable differences in the structure of control and treated tissue, although granulocytes could be more frequently distinguished in close proximity to epithelium in LPS and LTA-treated explants (Fig. 2C–E). We also evaluated tissue composition by flow cytometry (Fig. 3A) in order to check if treatments with LPS or LTA could lead to a reduction of viable leukocytes, granulocytes and macrophages. As described in the Fig. 3B–C, no differences were observed in the percentage of these cells upon tissue treatments with LPS and LTA when compared to controls.

**Fig. 4:**
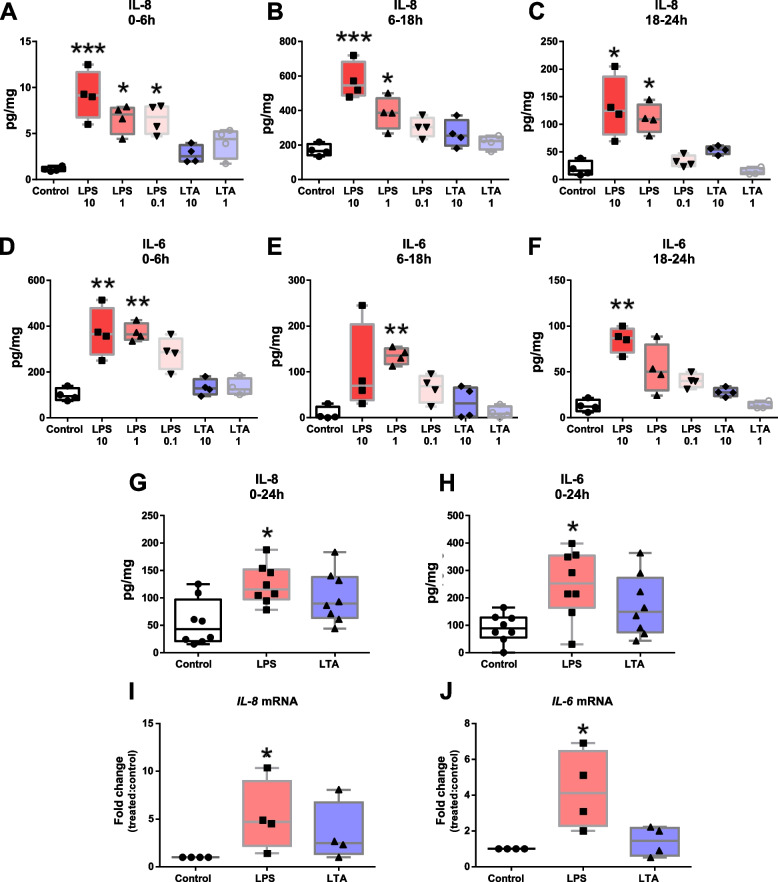
Inflammatory response of teat explants to TLR-agonists. **A–F** Time-course and dose-dependent analysis of IL-8 and IL-6 release by ELISA. Explants were exposed to LPS (10, 1 and 0.1 µg/mL) or LTA (10 and 1 µg/mL) and cytokine release was analysed at different intervals post stimulation (0–6 h, 6–18 h and 18–24 h). **G–H** Explants were treated with LPS (1 µg/mL) or LTA (10 µg/mL) and cytokine release was analysed 24 h post-stimulation. **I–J** Relative *IL-8* (**I**) and *IL-6* (**J**) mRNA expression in teat explants treated with LPS (1 µg/mL) and LTA (10 µg/mL) for 6 h. Box-plots show data from 4 independent replicates (4 animals, 1 teat/animal), except for **G** (8 independent replicates (8 animals, 1 teat/animal)). Data were analysed by Kruskal–Wallis test coupled to Dunn’s multiple comparisons test. Asterisks indicate that values differ from control (^*^P < 0.05, ^**^P < 0.01 and ^***^P < 0.001)

In order to check the biological relevance of our model, we next set out to verify if major TLR-agonists of Gram-positive and Gram-negative bacteria could trigger a proinflammatory response of teat tissue stimulated ex vivo. For this, a kinetics and dose-dependent experiment was carried out to establish the best conditions to evaluate tissue response. As shown in Fig. 4A–F, explants treated with 10 and 1 μg/mL of LPS produced significantly higher amounts of IL-8 and IL-6 than did control explants at any of the analysed time points. Interestingly, treatments with the same amounts of LTA led to IL-8 and IL-6 release but differences were not statistically significant when compared to controls. To confirm these observations, explants were treated with 1 and 10 μg/mL of LPS and LTA, respectively, and cytokine release was verified after 24 h incubation. Figure 4G–H shows that LPS but not LTA led to a significantly increased accumulation of both cytokines. The same response pattern was observed for *IL-8* and *IL-6* mRNAs (Fig. 4I–J).

### *E. coli* triggers stronger inflammatory response in teat than *S. aureus*

**Fig. 5:**
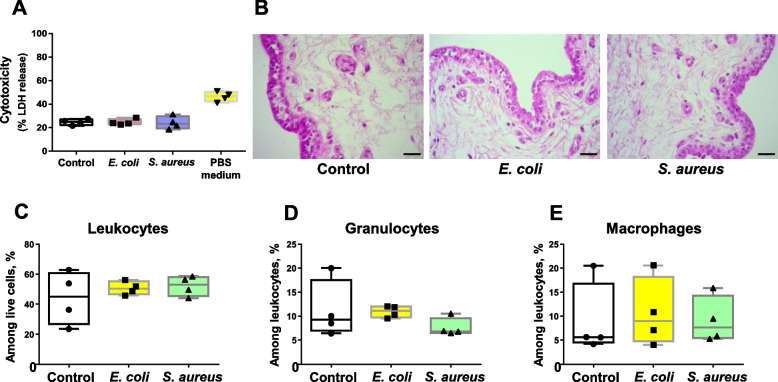
Tissue integrity and cellularity upon stimulation with bacteria. **A** Evaluation of cytotoxicity by lactate dehydrogenase (LDH) release assay. Explants were stimulated with heat-killed *Escherichia coli* and *Staphylococcus aureus* for 24 h. Complete medium alone and DPBS (nutrient starvation) were used as negative and positive controls, respectively. Box-plot shows data from 4 independent replicates (4 animals, 1 teat/animal). **B** Histological analysis of explants stimulated as described in **A**. Black bars = 20 µm. **C–E** Analysis of cell populations by flow cytometry. Percentages of leukocytes (**C**), granulocytes (**D**) and macrophages (**E**). Box-plots show data from 4 independent replicates (4 animals, 1 teat/animal). Data were analysed by Kruskal–Wallis test coupled to Dunn’s multiple comparisons test. Asterisks indicate that values differ from control (^*^P < 0.05, ^**^P < 0.01 and ^***^P < 0.001)

To further explore the relevance of our model, we evaluated the inflammatory responses triggered in bovine teat upon contact with heat-killed *E. coli* and *S. aureus.* Firstly, Fig. 5A shows that none of the pathogens compromised tissue viability. Then, microscopic examination demonstrated that tissue architecture is preserved after exposure to both *E. coli* and *S. aureus,* although granulocytes could be more frequently observed close to the epithelial cells layer (Fig. 5B). In addition, flow cytometry analysis confirmed the absence of major tissue cellularity alterations as the percentages of viable leukocytes, granulocytes as well as macrophages were comparable to controls after exposure to bacteria (Fig. 5D–E).

**Fig. 6:**
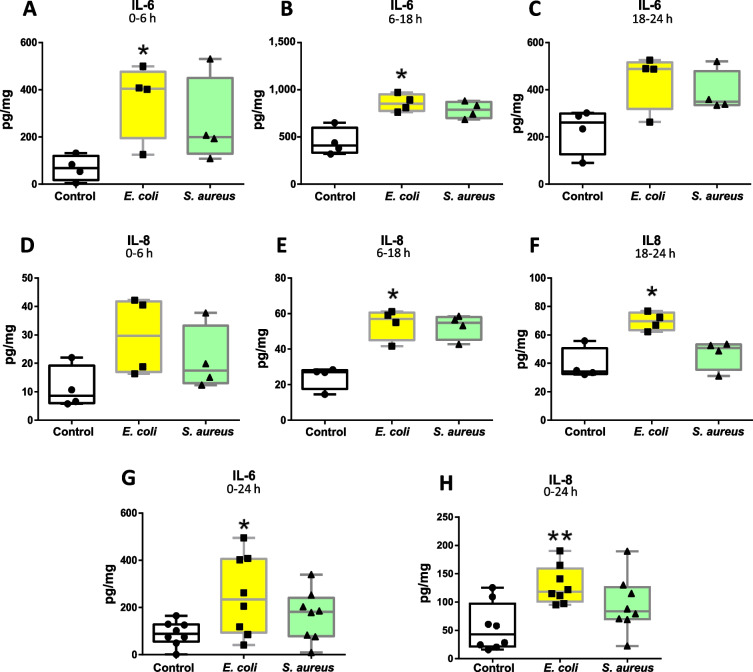
Pro-inflammatory cytokine release by teat explants in response to *Escherichia coli* and *Staphylococcus aureus.* **A–F** Time-course analysis of IL-8 and IL-6 release by ELISA. Explants were stimulated with heat-killed *E. coli* and *S. aureus* for 24 h and cytokine production was analysed at different intervals post stimulation (0–6 h, 6–18 h and 18–24 h). **G–H** Cytokine production analysed 24 h post-stimulation. Box-plots show data from 4 independent replicates (4 animals, 1 teat/animal), except for **G** and **H** (8 independent replicates (8 animals, 1 teat/animal)). Data were analysed by Kruskal–Wallis test coupled to Dunn’s multiple comparisons test. Asterisks indicate that values differ from control (^*^P < 0.05 and ^**^P < 0.01)

To better understand these results, we evaluated the impact of both pathogens on the induction of proinflammatory responses at both protein and transcriptional levels. Figure 6A–C shows that *E. coli* induced higher accumulation of IL-6, especially at the first hours after pulsing (0–18 h interval), whereas IL-8 release was observed to be significantly higher at later time intervals (Fig. 6D–F). In addition, cytokine release after 24 h exposure confirmed that *E. coli* but not *S. aureus* induce the release of proinflammatory cytokines by teat tissue (Fig. 6G–H).

**Fig. 7:**
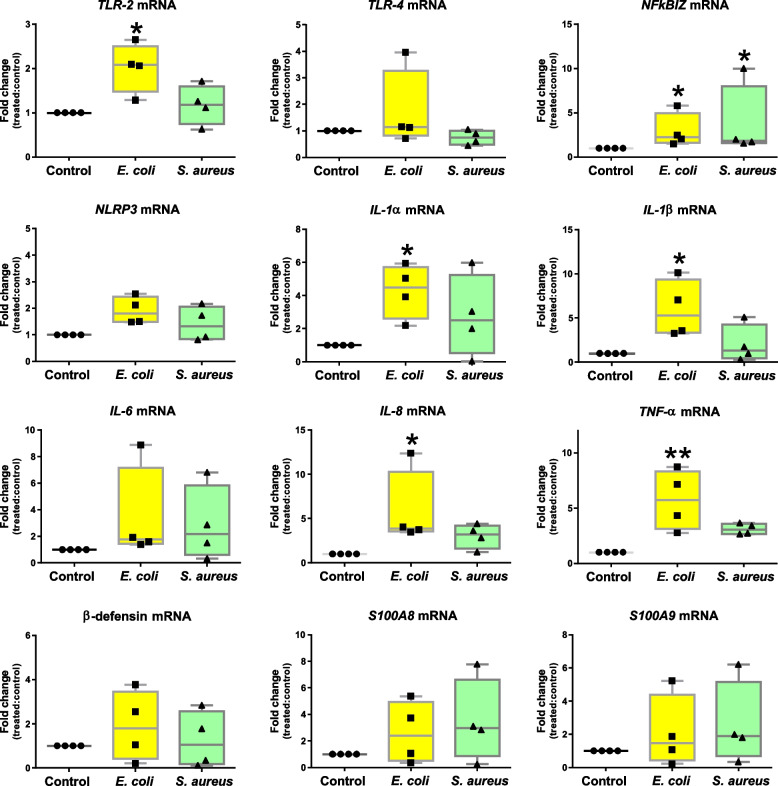
Relative mRNA expression of pro-inflammatory genes in teat explants upon stimulation with *Escherichia coli* and *Staphylococcus aureus.* Explants were stimulated with heat-killed *E. coli* and *S. aureus* for 24 h. Box-plots show data from 4 independent replicates (4 animals, 1 teat/animal). Data were analysed by Kruskal–Wallis test coupled to Dunn’s multiple comparisons test. Asterisks indicate that values differ from control (^*^P < 0.05 and ^**^P < 0.01)

The relative expression analysis of twelve genes coding for mediators of inflammation demonstrated that *E. coli* led to the upregulation of *TLR-2*, *NFkBIZ*, *IL-1α*, *IL-1β*, *IL-8* and *TNF-α*. Of note, *NFkBIZ* was the only gene differently regulated after stimulation with both *E. coli* and *S. aureus* (Fig. 7)*.*

### Frozen teat explants respond to ex vivo stimulation

**Fig. 8:**
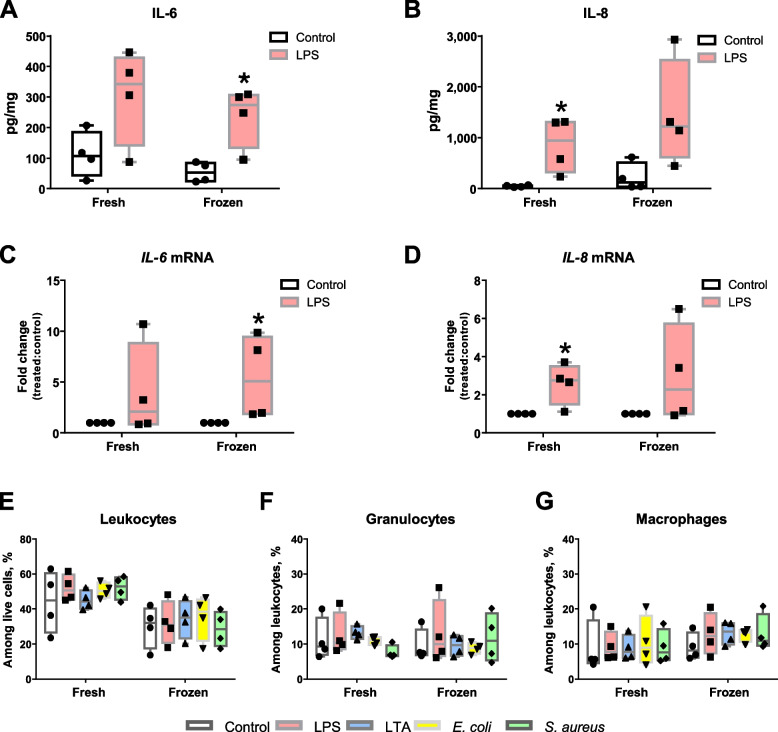
Analysis of fresh and frozen explants to treatments with TLR-agonists and bacteria. **A–B** Evaluation of IL8 and IL6 release by ELISA. Teat explant were frozen at −80 ºC in 10% DMSO FBS. After thawing, explants were stimulated with LPS (1 µg/mL) or complete medium alone (control) for 24 h. Fresh counterparts from the same teat were used for comparison. **C–D** Relative *IL-6* and *IL-8* mRNA expression in explants as in **A** and **B**. **E**–**G** Analysis of cell populations by flow cytometry. Explants were treated with LPS (1 µg/mL), LTA (10 µg/mL), heat-killed *E. coli* or *S. aureus*. Complete medium alone was used as control. Percentages of leukocytes (**E**), granulocytes (**F**) and macrophages (**G**). Box-plots show data from 4 independent replicates (4 animals, 1 teat/animal). Data were analysed by Kruskal–Wallis test coupled to Dunn's multiple comparisons test. Asterisks indicate that values differ from control (^*^P < 0.05)

Lastly, we verified if our ex vivo model could be applied to cryopreserved samples. Fresh and frozen explants from the same teats were stimulated and their capacity to mount an inflammatory response was checked by ELISA and qPCR. As described in Fig. 8A–B, frozen and fresh samples released similar amounts of IL-6 and IL-8 after LPS treatment for 24 h. Similar results were also observed when the expression of *IL-6* and *IL-8* encoding genes was evaluated (Fig. 8C–D). Additionally, the analysis of fresh and frozen tissue cellularity by flow cytometry revealed that frozen samples showed slightly lower percentages of live leukocytes (Fig. 8E). Nevertheless, the percentage of granulocytes and macrophages among live leukocytes was the same in both fresh and defrosted samples and did not change after pulsing with TLR-agonists nor bacteria.

## Discussion

This study describes an accessible ex vivo model to explore immune mechanisms that take place in the bovine teat, as an alternative to in vivo experimentation. It is based on the use of punch-excised teat tissue explants that can be maintained in culture for at least 24 h. We show that this approach yields tissue samples keeping typical teat microscopic architecture and functional features. To our best knowledge, this is the first description of teat immune response to mastitis-causing bacteria based on ex vivo tissue stimulation.

Most of the previous reports using explant models to study the MG relied on tissue low thickness cuts from the mammary secretory parenchyma [17, 18, 31]. Nevertheless, as mastitis-causing pathogens invade the udder via the teat canal, ex vivo analysis of this part of the MG provides a valuable view of the early response to infection. The punch-excised tissue model has been previously used to study the response of bovine endometrium to infection [32] but not to study the response of the MG during mastitis. This approach has several advantages over other in vitro or ex vivo infection models.

Tissue sampling with surgical punches gives the possibility of obtaining multiple explants from the same teat, which enables the stimulation of replicates from a common sample. Another advantage is the possibility to keep a sizeable amount of supporting connective tissue with a vascular compartment, allowing vascular leukocytes to take part in the response to stimuli.

A previous study employed explants excised from the teat sinus (cubic fragments of 2 mm edge) including the epithelium lining but removing the main part of connective tissue [33]. In our study, by using surgical punches, the surface of the epithelium is standardized, and by retaining a significant portion of subepithelial connective tissue, the epithelium benefits from a more preserved physiological environment than in other in vitro or ex vivo models. Keeping the tissue architecture, with normal spatial arrangement of cells and interactions with extracellular matrix, enables the model to yield more biologically relevant responses to stimuli.

Compared to sliced or chopped tissue explants, the punched explants are likely to release less damage-associated molecular patterns (DAMPs) that are generated in response to tissue disruption. In a study of bovine endometrium immune response to bacterial challenge, intact explants produced less cytokines than chopped explants [32]. This observation is possibly related to the fact that the exposure to TLR agonists is more physiological and less DAMPs are generated in intact explants. Low baseline production of markers of inflammation and less intense response to bacterial stimuli than with cut out tissue may then result from both conservation of the physiological tissue architecture and a reduced combination of DAMPs and microbe-associated molecular patterns (MAMPs) stimuli.

TLR-agonists recognition by Toll-like receptors (TLR) triggers early immune defence mechanisms by initiating a cascade of downstream events leading to the release of antimicrobial molecules, chemokines and cytokines from host cells [8, 20, 34]. In this study, we show that LPS-treated explants carry out stronger inflammatory response when compared to LTA, marked by an increased production of IL-6 and IL-8, at both mRNA and protein levels. These results corroborate previous studies reporting that LPS but not LTA leads to the upregulation of IL-6 and IL-8 encoding genes in MG secretory tissue [17, 18].

In line with this, *E. coli* induced higher cytokine release and the up-regulation of more pro-inflammatory in teat than *S. aureus. E. coli* has been widely reported to cause stronger inflammatory response in the bovine MG when compared to *S. aureus* [6, 8, 9, 21]. Since mastitis-causing bacteria initially encounter teat mucosa upon MG invasion, it is tempting to deduce that such differences could be associated to a better capacity of teat cells to recognise *E. coli* PAMPs and initiate innate immunity mechanisms. TLR-4 and TLR-2 have been associated to *E. coli* and *S. aureus* recognition, respectively. However, evidence indicates that these receptors can be activated by both pathogens [8, 20, 35]. Although *E. coli*-inoculation in bovine udder has been reported to cause a massive upregulation of TLR-2 and TLR-4 in mammary secretory tissue [36], we found out that TLR-2 but not TLR-4 was upregulated in teat explants exposed to *E. coli,* suggesting possible differences in the capacity of udder compartments to respond to infection. In agreement with Petzl et al. [36], our results also demonstrated that *S. aureus* do not induce the upregulation of TLR-4 nor TLR-2 in MG tissue.

NFKBIZ is a key regulator of the NF-κB inflammation pathway considered to be critical for mastitis pathogenesis [37, 38]. Indeed, NFκBIZ has been identified in bovine mammary epithelial cells as a differentially expressed gene continuously upregulated in response to *E. coli* and *S. aureus* [39]. Interestingly, we uncovered *NFκBIZ* as the sole proinflammatory gene differentially regulated after teat stimulation with *S. aureus*. As NF-kB activation relies on PAMP recognition by TLR and other pattern recognition receptors (PRR), it can be deduced that teat mammary cells sense *S. aureus* but fail to trigger robust inflammatory response. Reinforcing this hypothesis, genes encoding for major orchestrators of immune mechanisms to clear bacteria, such as *IL-1α*, *IL-1β*, *IL-8* and *TNF-α*, were upregulated in teat upon stimulation with *E. coli* but not with *S. aureus.* The same observation has been previously described in bovine mammary epithelial cells [35] and udder cistern tissue of experimentally infected cows [40].

NLRP3 inflammasome is a cytosolic signalling complex of the innate immune system also reported as an important mediator of host defence against bacteria [41–43]. In this study, we show that *E. coli* and *S. aureus* increase the expression of NLRP3 in bovine teat explants, but not significantly, which differs from findings in bovine mammary epithelial cells infected with *S. aureus* [42] or pulsed with *E. coli* LPS [43]. Similarly, we observed that β-defensin expression was not significantly altered in teat explants stimulated with *E. coli* nor *S. aureus*. β-defensins are antimicrobial peptides previously associated to bovine innate defence against mastitis [44], their encoding genes being highly expressed in mammary quarters infected with *S. aureus* [45]. *E. coli* and LPS have been reported to induce β-defensin upregulation in primary bovine mammary epithelial cells [46]. Such discrepancies strengthen the idea that the nature of innate mechanisms triggered in the teat could differ from other mammary compartments, since the mentioned observations were based on analysis of infected parenchyma and primary or stable cell lines derived from secretory tissue. Nevertheless, differences due to the time points analysed in this and the cited studies should not be excluded.

High levels of proteins S100A8 and S100A9 play a decisive role in the development of inflammation. After infection with bacteria, neutrophils, macrophages and monocytes secrete S100A8/A9 to modulate inflammatory processes characterized by the production of cytokines, reactive oxygen species (ROS) and nitric oxide (NO) [47]. Previous observations on goat MG pointed out the production of S100A8 after intramammary infusion of LPS [48]. In this study, bovine teat explants pulsed with *E. coli* or *S. aureus* do not show significant changes in S100A8/A9 regulation and this could be associated with the absence of changes in tissue cellularity intrinsic to our ex vivo approach.

## Conclusions

This study provides an ex vivo model to shed light on the immune response mechanisms carried out in bovine teat in response to infections. Our experimental strategy enabled us to demonstrate, in compliance with the 3Rs principles, that teat response to two major mastitis-causing pathogens differs. We also showed that this approach can be adapted to cryopreserved tissue, facilitating its application to large scale and long-term studies. Every in vitro or ex vivo model has limitations [8]. However, due to its features and ease of use, we believe that the punch-excised explant model is an improvement over most others. It lends itself to further development to study different aspects of the immune response of MG to infections and might be of great use in future research on mastitis.

## Supplementary Information

**Additional file 1: Fig. S1.** Comparison of tissue digestion methods for flow cytometry analysis. Part A.

**Additional file 2: Fig. S2.** Comparison of tissue digestion methods for flow cytometry analysis. Part B.

**Additional file 3: Table S1.** List of primers used for qPCR analysis.

## Data Availability

Not applicable.

## Abbreviations

DAMPs: Damage-associated molecular patterns
H&E: Haematoxylin and eosin
LDH: Lactate dehydrogenase
LPS: Lipopolysaccharide
LTA: Lipoteichoic acid
MAMPs: Microbe-associated molecular patterns
MG: Mammary gland
NO: Nitric oxide
PRR: Pattern recognition receptors
ROS: Reactive oxygen species
TLR: Toll-like receptor
3Rs: Reduction, refinement and replacement
