# Supplementary figures and images for: Punch-excised explants of bovine mammary gland to model early immune response to infection
Source: J Anim Sci Biotechnol. 2023 Jul 7;14:100. doi: 10.1186/s40104-023-00899-0 (PMC10326946; doi:10.1186/s40104-023-00899-0)

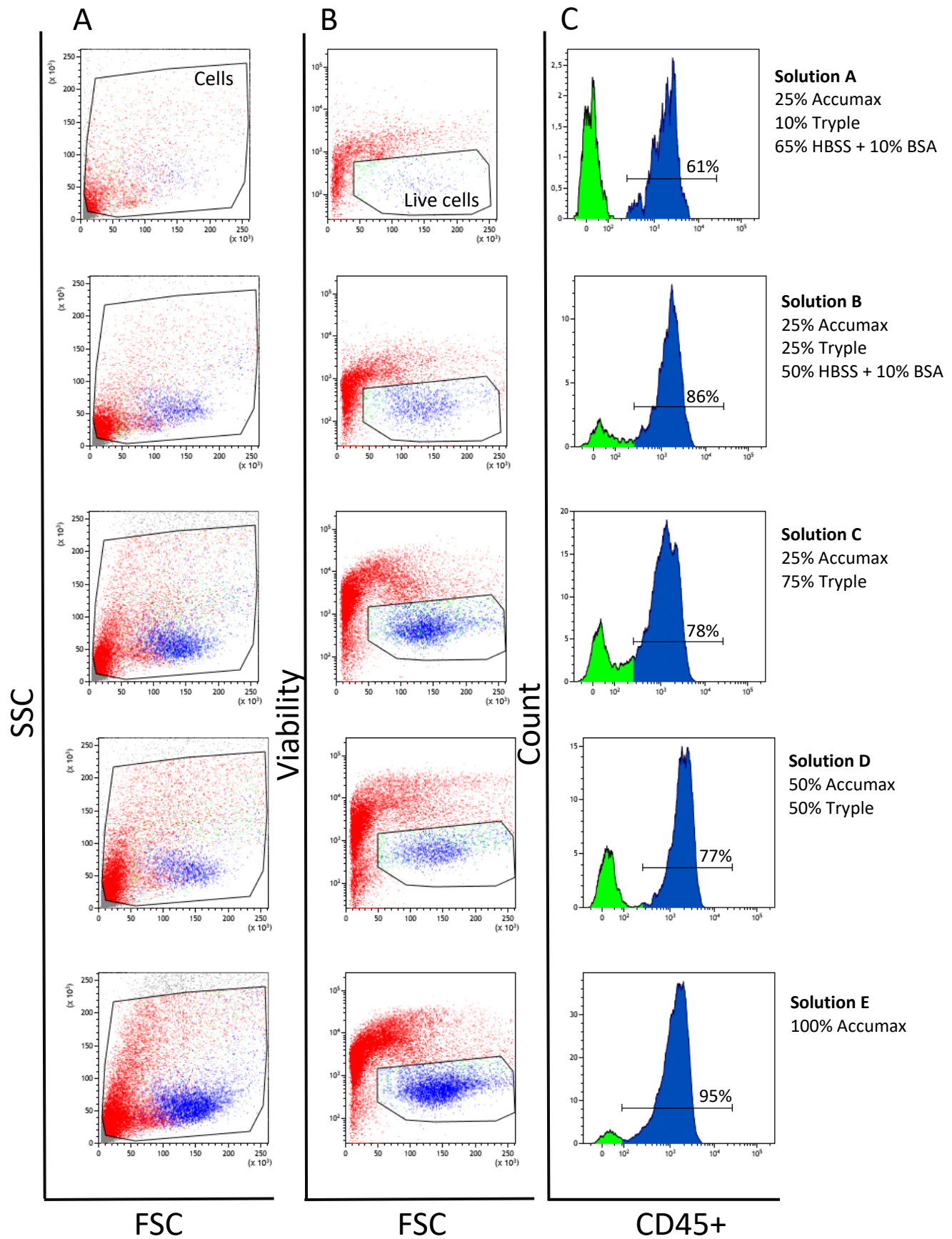

Supplement: Supplementary file 1 — Additional file 1: Fig. S1. Comparison of tissue digestion methods for flow cytometry analysis. Part A. [file 40104_2023_899_MOESM1_ESM.pdf]

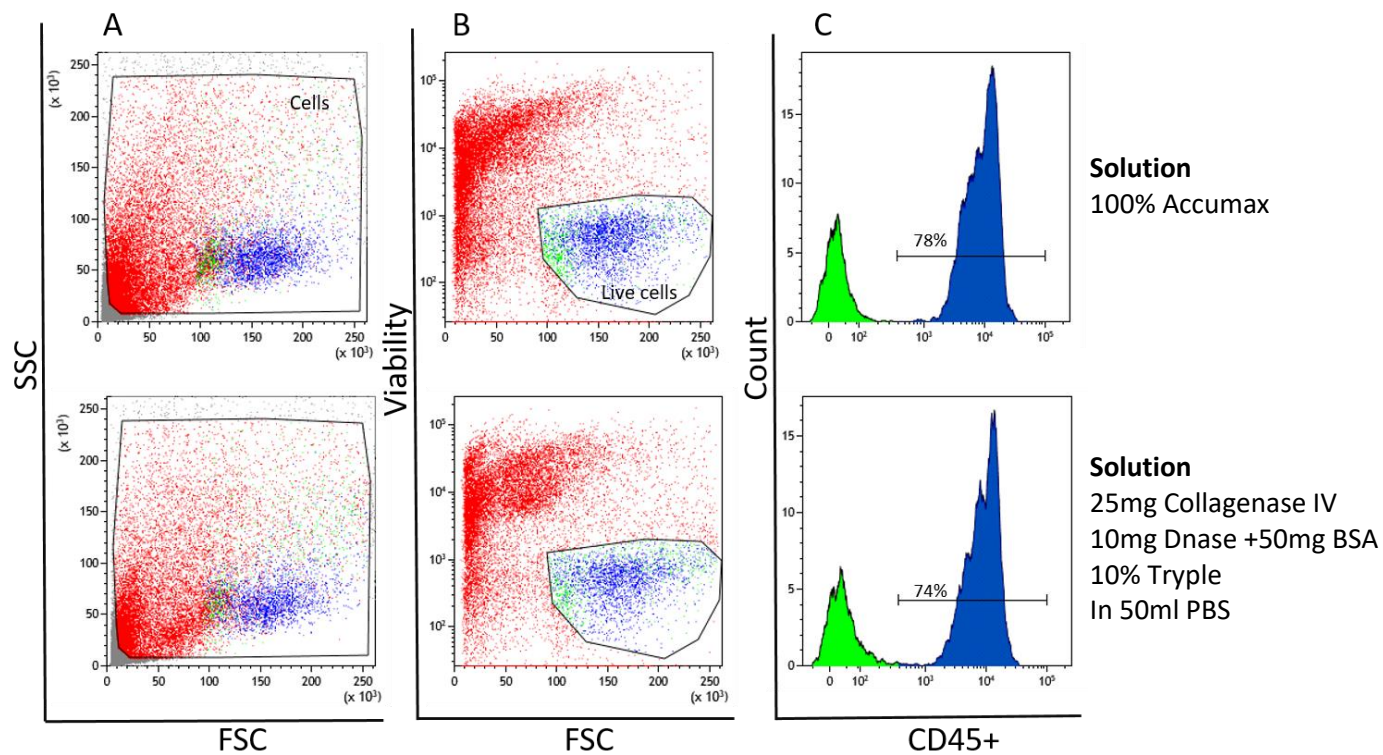

Supplement: Supplementary file 2 — Additional file 2: Fig. S2. Comparison of tissue digestion methods for flow cytometry analysis. Part B. [file 40104_2023_899_MOESM2_ESM.pdf]
