# Supplementary material for: Punch-excised explants of bovine mammary gland to model early immune response to infection
Source: J Anim Sci Biotechnol. 2023 Jul 7;14:100. doi: 10.1186/s40104-023-00899-0 (PMC10326946; doi:10.1186/s40104-023-00899-0)
Supplement: Supplementary file 3 — Additional file 3: Table S1. List of primers used for qPCR analysis. [file 40104_2023_899_MOESM3_ESM.pdf]

Table S1 Sequences of primers used in this study. Reference genes used to calculate ΔCT are indicated in the gray boxes

GENE ID	Reference Sequences	Forward	Reverse
ACTB	NM_173979.3	ACGGGCAGGTCATCACCATC	AGCACCGTGTTGGCGTAGAG
GAPDH	NM_001034034.2	GGCATCGTGGAGGGACTTATG	GCCAGTGAGCTTCCCGTTGAG
PPIA	NM_178320.2	TCCGGGATTTATGTGCCAGGG	GCTTGCCATCCAACCACTCAG
TLR2	NM_174197.2	ACTGGGTGGAGAACCTCATGGTCC	ATCTTCCGCAGCTTACAGAAGC
TLR4	NM_174198.6	GCATGGAGCTGAATCTCTAC	CAGGCTAAACTCTGGATAGG
NLRP3	NM_001102219.1	CTCAGTGGCAATACCCTGGG	AGCACTGTCCCAACCACAAT
NFkB	NM_174726.1	GGCGGAAATCCAATGAGCAC	TATGCAGGAACGTGTCACCA
TNFα	NM_173966.3	TCTTCTCAAGCCTCAAGTAACAAGC	CCATGAGGGCATTGGCATA
IL1α	NM_174092	CTGAAGAAGAGACGGTTGAG	ATGCATTCCTGGTGGATGAC
IL1β	NM_174093.1	CTCTCACAGGAAATGAACCGAG	GCTGCAGGGTGGGCGTATCACC
IL6	NM_173923.2	TGCTGGTCTTCTGGAGTATC	GTGGCTGGAGTGGTTATTAG
CXCL8	NM_173925.2	TGAAGCTGCAGTTCTGTCAAG	TTCTGCACCCACTTTTCCTTGG
DEFB5	NM_001130761	TCGTGCTCCTCTTCCTAGTC	GGCACGAGATCGGAATACAG
S100A8	NM_001113725	CTCCCTGATTGACGTCTACC	TCCAGGCCCACCTTTATCAC
S100A9	NM_001046328	TGACACCCTGATCCAGAAAG	GCCACCAGCATAATGAACTC
